# Myocardial iron overload in sickle/thalassemia patients of Italian origin

**DOI:** 10.1186/1532-429X-15-S1-E106

**Published:** 2013-01-30

**Authors:** Antonella Meloni, Giovan Battista Ruffo, Petra Keilberg, Domenico D'Ascola, Alessandra Quota, Claudio Ascioti, Vincenzo Positano, Cristina Salvatori, Letizia Gulino, Massimo Lombardi, Alessia Pepe

**Affiliations:** 1 CMR Unit, Fondazione G.Monasterio CNR-Regione Toscana and Institute of Clinical Physiology, Pisa, Italy; 2 ARNAS Ospedale Civico, Palermo, Italy; 3 U.O. Microcitemie, A.O. "Bianchi-Melacrino-Morelli", Reggio Calabria, Italy; 4 Serv. Talassemia, Osp. "V. Emanuele III", Gela, Italy; 5 Struttura Complessa di Cardioradiologia, P.O. "Giovanni Paolo II", Lamezia Terme, Italy; 6 Fondazione G.Monasterio CNR-Regione Toscana, Pisa, Italy

## Background

Sickle-thalassemia is an inherited hemoglobin disorder resulting from the combined heterozygosity for sickle-cell and β-thalassemia genes. Myocardial iron overload in patients with sickle-thalassemia has been poorly studied; however, a report has shown no evidence of cardiac iron in a small group (n=10) of multitransfused Arab patients. The current study aims to further evaluate cardiac iron overload in a larger group of Italian patients using a T2* multislice approach and explore its correlation with transfusions, age and sex.

## Methods

Fifty-nine sickle-thalassemia patients (29 males, mean age 35.6±14.1 years), enrolled in the MIOT (Myocardial Iron Overload in Thalassemia) network were considered. Three parallel short-axis views of the left ventricle were acquired and analyzed with a dedicated software (HIPPO MIOT) providing the T2* value on each of 16 segments as well as the global T2* value averaged over all segmental T2* values and the T2* value in the mid-ventricular segment averaged over the mid-anterior and the mid-inferior septum.

## Results

We found 55 (93%) patients with all 16 segmental T2* values normal (>20 ms). Of the 4 patients with abnormal segmental T2* values, all showed an heterogeneous MIO (some segments with T2* values >20 ms and other segments with T2* values <20 ms) and none showed an homogeneous MIO (all segment with T2* values <20 ms). Out of the 4 patients with heterogeneous MIO, only one had a global T2* global <20 ms.

The mean global heart T2* value was 34.4±6.2 ms.

We did not find significant differences among sickle-thalassemia regularly (N=20), sporadically (N=32) and no transfused (N=7) in the T2* global value (33.4±7.3 ms versus 35.5±5.4 ms versus 32.4±6.3 ms; P=0.425).

On linear regression analysis, there was a statistically significant positive correlation between global T2* and age but with poor linearity (R=0.368; P=0.004).

The global T2* value was not significant different between males and females (35.6±4.9 ms versus 35.2±7.2 ms; P=0.118).

## Conclusions

In respect of myocardial iron deposition, the sickle/thalassemia patients are similar to patients with homozygous SCD for which iron overloading is relatively rare.

## Funding

The MIOT project receives "no-profit support" from industrial sponsorships (Chiesi and Apotex). This study was also supported by: "Ministero della Salute, fondi ex art. 12 D.Lgs. 502/92 e s.m.i., ricerca sanitaria finalizzata anno 2006" e "Fondazione L. Giambrone".

